# Red Cell Distribution Width and Mortality in Patients Undergoing Percutaneous Coronary Intervention

**DOI:** 10.3390/biomedicines10010045

**Published:** 2021-12-26

**Authors:** Min-Tsun Liao, Chao-Lun Lai, Ting-Chuan Wang, Jou-Wei Lin, Yi-Lwun Ho, K. Arnold Chan

**Affiliations:** 1 Department of Internal Medicine, National Taiwan University Hospital Hsin-Chu Branch, Hsin-Chu 300, Taiwan; mintsunliao@ntu.edu.tw; 2 Department of Internal Medicine, College of Medicine, National Taiwan University, Taipei 100, Taiwan; Y00039@ms1.ylh.gov.tw (J.-W.L.); ylho@ntu.edu.tw (Y.-L.H.); 3 Institute of Epidemiology and Preventive Medicine, College of Public Health, National Taiwan University, Taipei 100, Taiwan; 4 Health Data Research Center, National Taiwan University, Taipei 100, Taiwan; 118678@ntuh.gov.tw (T.-C.W.); kachan@ntu.edu.tw (K.A.C.); 5 Cardiovascular Center, National Taiwan University Hospital Yunlin Branch, Douliu City 640, Taiwan; 6 Department of Internal Medicine, National Taiwan University Hospital, Taipei 100, Taiwan; 7 Graduate Institute of Oncology, College of Medicine, National Taiwan University, Taipei 100, Taiwan

**Keywords:** red cell distribution width, percutaneous coronary intervention, mortality

## Abstract

Red cell distribution width (RDW) can effectively predict prognosis in coronary artery disease (CAD) patients following percutaneous coronary intervention (PCI). There is currently no relevant research to demonstrate a linear or non-linear association between RDW and mortality. This is a multi-center, retrospective cohort study, with data collected from 2006 to 2017. Source data included electronic medical records of the Integrated Medical Database of National Taiwan University Hospital, and health insurance claims from the National Health Insurance Administration. Patients were stratified into five groups according to RDW values (13.4%, 14.1%, 14.8%, and 15.9%). Multivariable logistic and Cox regression analyses were used to determine 1-year all-cause and cardiovascular (CV) mortalities. Data of 10,669 patients were analyzed and those with the lowest RDW (≤13.3%) served as the reference group. The adjusted odds ratios (ORs) of 1-year all-cause mortality from the second to fifth RDW group were 1.386, 1.589, 2.090, and 3.192, respectively (*p* for trend < 0.001). The adjusted ORs of 1-year CV mortality were 1.555, 1.585, 1.623, and 2.850, respectively (*p* for trend = 0.015). The adjusted hazard ratios (HRs) of 1-year all-cause mortality were 1.394, 1.592, 2.003, and 2.689, respectively (*p* for trend = 0.006). The adjusted HRs of 1-year CV mortality were 1.533, 1.568, 1.609, and 2.710, respectively (*p* for trend = 0.015). RDW was an independent predicting factor and had a linear relationship with the 1-year all-cause and CV mortalities in patients undergoing PCI. Thus, RDW may be a clinically useful parameter to predict the mortality in those patients.

## 1. Introduction

Red cell distribution width (RDW) is the distribution of erythrocyte sizes derived from automated hematology analyzers, which can be used as a reliable index for anisocytosis [1]. RDW is calculated as a percentage value obtained by dividing a standard deviation of the erythrocyte size distribution by the mean red cell volume [2]. The normal value is between 11–15% [3], but it can be affected by inter-instrument differences including impedance or optical techniques and inter-laboratory differences [4]. In addition, RDW is affected by blood transfusion [5], acute or chronic heart failure [6], autoimmune disease [7] and neoplasms [8]. Currently RDW is mainly used as a differential diagnosis of microcytic or normocytic anemia. However, recent research has found that RDW can be used as an index for risk stratification of cardiovascular (CV) diseases [9], and it can also improve the effectiveness of mortality stratification for patients with high-risk critical illness [10]. RDW may also be applicable to various other populations. Whether RDW is a specific marker of risk-stratification still needs to be evaluated.

Coronary artery disease (CAD) is caused by coronary artery atherosclerosis and endothelial hyperplasia [11]. Plaque rupture of the coronary artery can cause thrombosis, and block the coronary blood flow, which leads to myocardial infarction (MI) and necrosis [11]. The diagnostic standard for CAD is coronary angiography [12,13]. In addition to the control of risk factors such as hypertension, diabetes, hyperlipidemia, and smoking, invasive percutaneous coronary intervention (PCI) for CAD is the standard treatment [12,13]. With the evolution of technology, such as the development of balloon angioplasty, bare-metal stents and drug-eluting stents, PCI can effectively reduce the mortality rate in patients with ST elevation MI [14]. Many studies including meta-analysis showed that RDW can effectively predict all-cause mortality and CV mortality in patients following PCI [15,16,17,18]. However, because of the insufficient sample size, previous studies only use one specific value of RDW to analyze the differences between the lower and higher groups, and the cut-off values of RDW identified in studies in the meta-analysis are also different. There is no relevant research to demonstrate whether the relationship between RDW and mortality is a linear or non-linear association. The purpose of this study is to conduct a large-scale multi-center retrospective study, spanning more than 10 years, to analyze the role of RDW in the prediction of 1-year all-cause mortality and CV mortality in CAD patients undergoing PCI.

## 2. Materials and Methods

### 2.1. Study Design and Patients Selection

This study is a multi-center and retrospective cohort study. Eligible patients were considered to meet the following criteria: (1) age ≥ 20 years; (2) patients who received PCI from 2006 through 2017. Exclusion criteria were: (1) no RDW data when admission; (2) patients who died during the index hospitalization. For patients hospitalized more than once during the study period, the first hospitalization was used as the index hospitalization. Then, patients were stratified into 5 groups, according to RDW values on admission [10].

### 2.2. Data Sources

This study used the electronic medical records incorporated into the Integrated Medical Database of National Taiwan University Hospital (NTUH-iMD), including the data from National Taiwan University Hospital (NTUH) from 2007 onward, the NTUH Yunlin Branch from February 2014 onward, and the NTUH Hsinchu Branch from November 2014 onward. In addition, health insurance claims data from Taiwan National Health Insurance (NHI) Administration and the national mortality data were utilized. Taiwan has implemented universal health insurance since 1995 [19], and approximately 97% of citizens have participated in the insurance [20]. Relevant clinical data including personal identification number, date of birth, gender, date of outpatient visit, hospital admission and discharge, diagnostic codes, procedure codes, and drugs administered, are available from the NHI for research. Through the end of 2015, the International Classification of Diseases, Ninth Revision, Clinical Modification (ICD-9-CM) system was used as the diagnostic codes and the International Classification of Diseases, Tenth Revision, Clinical Modification (ICD-10-CM) system has been used since 2016.

In this study, patients with PCI in the inpatient procedure were identified from the NTUH-iMD. Then, the patients’ identification number was used to link the database of NHI Administration to obtain background characteristics of patients and national mortality data to obtain the definite date of death. All linkages were carried out under guidelines specified by the Department of Statistics, Ministry of Health and Welfare and National Taiwan University Hospital to protect individuals’ privacy. This research was approved by Institutional Review Board of NTUH (201802076RINB), and all research methods and data analysis were carried out in accordance with regulations.

### 2.3. Clinical Attributes and Outcomes Assessment

Baseline characteristics including age, sex, body mass index, type and total number of coronary stents, diagnosis of hospitalization, medications at discharge, and laboratory data of index hospitalization were ascertained from the NTUH-iMD. Previous medical history was assessed from both the NTUH-iMD and the Taiwan NHI claims database. The evaluation time period of the baseline characteristics was 18 months prior to the index hospitalization. In addition to comorbidity defined in the Elixhauser comorbidity index [21], we also defined hyperlipidemia as ICD9: 272.x; ICD10: E78.x, ischemic stroke as ICD9: 433.x1, 434.x1, 435.9x, 436.x, 437.1x, 437.9x; ICD10: I63.x, previous MI as ICD9: 410.x, 412.x; ICD10: I21.x, I22.x, I23.x, I25.2x, acute ST-elevation MI as ICD9: 410.0x-410.6x, 410.8x; ICD10: I21.0x-I21.3x, I22.0x, I22.1x, I22.8x, I22.9x, acute non-ST elevation MI as ICD9: 410.7x, 410.9x; ICD10: I21.4x, I21.9x, I21.Ax, I22.2x, unstable angina as ICD9: 411.x; ICD10: I20.0x, I23.7x, I24.0x, I24.8x, I24.9x, and stable angina as ICD9: 412.x, 413.x, 414.x; ICD10: I20.1x, I20.8x, I20.9x, I25.x. A patient with diagnosis codes of interest two or more times in the outpatient records during the 18-month baseline period, or once in the inpatient records, was defined as having a comorbidity of interest. The clinical outcomes of this study included the all-cause mortality and CV mortality (ICD9: 353.x-459.x; ICD10: I05.x-I99.x) at 6 months and 1 year after discharge from the index hospitalization, respectively.

### 2.4. Statistical Analyses

The first RDW value after admission was used to categorize the patients into five groups according to previously reported cut-off values (13.4%, 14.1%, 14.8%, and 15.9%) [10]. Mean and standard deviation were used to describe the continuous variables. Number and percent were used to present the categorical data. ANOVA and the chi-squared test were used to compare the characteristics between RDW groups. A multivariable logistic regression model, adjusting for the confounding variables, was used to compare the various clinical outcomes between RDW groups. A receiver operating characteristic (ROC) curve was plotted to determine the best cut-off value of RDW, according to the maximum value of Youden index [22]. A Cox proportional hazard regression analysis was also used for time-to-event analysis and the cumulative probabilities of all-cause or CV mortalities were estimated by the Kaplan–Meier curves. All analysis was performed using SAS software, version 9.4 (SAS Institute, Inc., Cary, NC, USA).

## 3. Results

### 3.1. Study Subjects

We identified 15,319 patients undergoing PCI from January 2006 to December 2017, 4405 patients had no RDW data during hospitalization and 245 patients died in the hospital, resulting in 10,669 survivors discharged from the hospitals after PCI; 8382 (78.6%) were men and 2287 (21.4%) were women, and the mean age was 65.4 ± 12.1 years (Supplementary Figure S1). We divided the patients into five groups, according to the first value of the RDW during the hospitalization. There were significant differences in basic characteristics between the RDW groups, including: age; sex; hemoglobin; estimated glomerular filtration rate; white blood cells; total cholesterol; triglyceride; body mass index; hypertension; hyperlipidemia; diabetes mellitus; current smoking status; previous MI previous ischemic stroke; peripheral vascular disease; chronic pulmonary disease; previous PCI; previous coronary artery bypass grafting surgery; admission diagnosis; and type of intervention. Moreover, they also used different medications, including: ticagrelor; amiodarone; diltiazem; dihydropyridine calcium channel blockers; angiotensin-converting enzyme inhibitors; loop diuretics; spironolactone; statins; oral antidiabetic drugs; insulin; and proton-pump inhibitors. The patients in higher RDW groups had lower hemoglobin and estimated glomerular filtration rate, and a higher rate of hypertension, diabetes, previous MI, previous ischemic stroke, previous coronary artery bypass grafting surgery, and myocardial infarction. Therefore, the patients in the higher RDW groups were sicker than patients in the lower RDW groups (Table 1).

### 3.2. Mortality Outcomes

The 6-month all-cause mortalities after discharge from the lowest RDW group to the highest RDW group were 1.23%, 2.89%, 4.50%, 7.70%, and 14.36%, respectively. The 6-month CV mortalities after discharge from the lowest RDW group to the highest RDW group were 0.60%, 1.29%, 2.12%, 2.44%, and 6.22%, respectively. The 1-year all-cause mortalities after discharge from the lowest RDW group to the highest RDW group were 2.28%, 4.90%, 8.21%, 13.09%, and 23.48%, respectively. The 1-year CV mortalities after discharge from the lowest RDW group to the highest RDW group were 1.00%, 2.36%, 3.53%, 4.62%, and 9.67%, respectively (Table 2).

### 3.3. Multivariable Logistic Regression Analysis

The lowest RDW (≤13.3%) group was used as the reference group. The crude odd ratios (ORs) of 1-year all-cause mortality from the second to fifth RDW group were 2.206 (95% confidence interval [CI] 1.705–2.855), 3.831 (95% CI 2.914–5.036), 6.455 (95% CI 4.925–8.459), and 13.146 (95% CI 10.305–16.771), respectively (*p* for trend = 0.004). The crude ORs of 1-year CV mortality from the second to fifth RDW group were 2.387 (95% CI 1.640–3.476), 3.615 (95% CI 2.404–5.436), 4.786 (95% CI 3.136–7.305), and 10.572 (95% CI 7.396–15.113), respectively (*p* for trend = 0.006) (Table 3).

After adjusting for all the variables listed in Table 1, the adjusted ORs of 1-year all-cause mortality from the second to fifth RDW group were 1.386 (95% CI 1.055–1.822), 1.589 (95% CI 1.180–2.138), 2.090 (95% CI 1.543–2.830), and 3.192 (95% CI 2.398–4.248), respectively (*p* for trend < 0.001). The adjusted ORs of 1-year CV mortality from the second to fifth RDW group were 1.555 (95% CI 1.053–2.295), 1.585 (95% CI 1.027–2.447), 1.623 (95% CI 1.023–2.574), and 2.850 (95% CI 1.883–4.312), respectively (*p* for trend = 0.015) (Table 3). The ORs of 6-month mortalities showed similar results, and are shown in Table 3.

### 3.4. Cox Proportional Hazards Regression Analysis and Kaplan–Meier Curves

The lowest RDW (≤13.3%) group was used as the reference group. The crude hazard ratios (HRs) of 1-year all-cause mortality from the second to fifth RDW group were 2.166 (95% CI 1.682–2.790), 3.681 (95% CI 2.823–4.800), 5.973 (95% CI 4.613–7.735), and 11.100 (95% CI 8.843–13.934), respectively (*p* for trend = 0.019). The crude HRs of 1-year CV mortality from the second to fifth RDW group were 2.375 (95% CI 1.636–3.446), 3.601 (95% CI 2.407–5.387), 4.792 (95% CI 3.162–7.264), and 10.378 (95% CI 7.328–14.698), respectively (*p* for trend = 0.006) (Supplementary Table S1).

**Figure 1:**
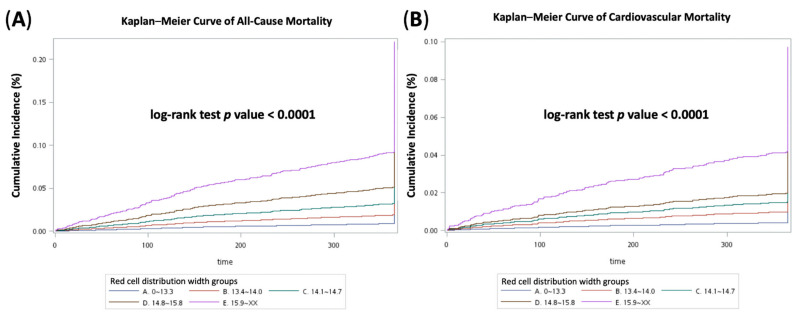
Kaplan–Meier curves of (**A**) 1-year all-cause mortality, and (**B**) 1-year cardiovascular mortality in all study patients.

The adjusted HRs of 1-year all-cause mortality from the second to fifth RDW group were 1.394 (95% CI 1.078–1.804), 1.592 (95% CI 1.208–2.099), 2.003 (95% CI 1.518–2.643), and 2.689 (95% CI 2.076–3.485), respectively (*p* for trend = 0.006). The adjusted HRs of 1-year CV mortality from the second to fifth RDW group were 1.533 (95% CI 1.049–2.240), 1.568 (95% CI 1.029–2.387), 1.609 (95% CI 1.031–2.509), and 2.710 (95% CI 1.825–4.026), respectively (*p* for trend = 0.015) (Supplementary Table S1). The cumulative all-cause death and CV death were estimated by Kaplan–Meier plots. The log-rank test showed significant differences between the groups (Figure 1).

### 3.5. Prediction Model and ROC Curves

**Figure 2:**
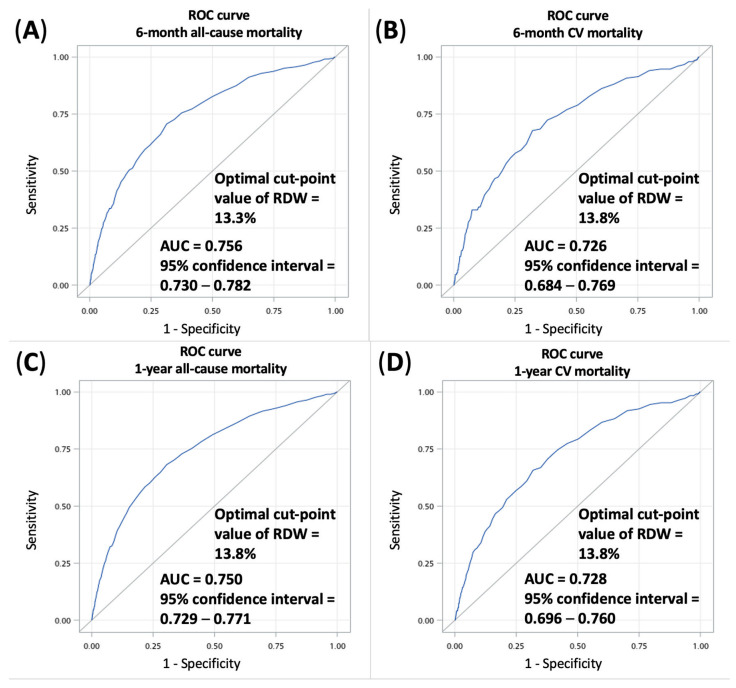
Receiver operating characteristic curves of (**A**) 6-month all-cause mortality; (**B**) 6-month cardiovascular mortality; (**C**) 1-year all-cause mortality; and (**D**) 1-year cardiovascular mortality in all study patients. Abbreviations: AUC, area under the receiver operating characteristic curve; CV, cardiovascular; ROC, receiver operating characteristic.

In order to predict the mortality outcomes using the all population of this study, the ROC curves from the logistic regression models predicting 6-month or 1-year all-cause or CV mortalities are presented in Figure 2. The AUC (area under the ROC curve) of 6-month and 1-year all-cause mortalities were 0.756 (95% CI 0.730–0.782) and 0.750 (95% CI 0.729–0.771), respectively. The AUC of 6-month and 1-year CV mortalities were 0.726 (95% CI 0.684–0.769) and 0.728 (95% CI 0.696–0.760), respectively. The optimal cut-point value of RDW was 13.3% for 6-month all-cause mortality and 13.8% for 6-month CV mortality, 1-year all-cause mortality and 1-year CV mortality. The adjusted OR of 6-month all-cause mortality in RDW ≥ 13.3% compared with RDW < 13.3% was 2.021 (95% CI 1.496–2.731). The adjusted OR of 1-year all-cause mortality in RDW ≥ 13.8% compared with RDW < 13.8% was 1.952 (95% CI 1.599–2.384). The adjusted ORs of CV mortality in RDW ≥ 13.8% compared with RDW < 13.8% were 1.919 (95% CI 1.314–2.802) at 6 months and 1.732 (95% CI 1.293–2.319) at 1 year, respectively (Supplementary Table S2).

## 4. Discussion

In this large-scale real-world study from a non-Caucasian population to evaluate the relationship between RDW and mortality in CAD patients undergoing PCI, we found that RDW was an independent factor associated with 1-year all-cause mortality and CV mortality in CAD patients undergoing PCI. RDW had a positive linear and dose–response relationship with 1-year all-cause mortality and CV mortality. The results indicated that the higher the RDW, the higher the all-cause mortality and CV mortality in patients with CAD undergoing PCI.

Previous studies have shown that RDW was associated with CV diseases [23] and RDW could be used as a parameter to predict all-cause mortality and CV mortality [24]. The possible mechanism is that RDW is an inflammation marker [25]. Inflammation can lead to abnormal bone marrow function, resulting in poor efficiency of red blood cell production, and also affects RBC membrane permeability, which causes reticulocytes to enter the peripheral blood circulation, and increases the proportion of immature RBC, leading to an increase in RDW [26]. Inflammatory markers are associated with mortality and major adverse CV events [27,28]. Therefore, RDW may be used as an indicator of mortality prediction. Another possible mechanism is that anisocytosis is directly involved in the pathogenic process of CV diseases [29]. The injury of the fibrous cap of the atherosclerosis plaque causes thrombosis, and red blood cells are entrapped in the atherosclerosis plaque of blood vessel wall. Once red blood cells are entrapped in the atherosclerosis plaque, a series of inflammatory reaction processes are activated and lead to CV diseases [29].

Pilling et al. reported a large-scale observational study including 240,477 healthy volunteers from UK Biobank [23]. The study demonstrated that those with higher RDW had higher all-cause mortality, with linear dose–response relationship, and also predicted new onsets of wide common conditions including incident coronary artery disease, heart failure, peripheral artery disease, atrial fibrillation, stroke and cancer. Aside from healthy volunteers, RDW has also been reported to be a robust predictor of the risk of all-cause mortality in older adults [30], the critically ill [10,31], non-CV critically patients [32], and those with heart failure [18,33], chronic kidney disease [34], and cancers including gliomas [35], and gastrointestinal cancers [36,37]. This evidence indicates that RDW may be a marker for predicting mortality from various diseases.

A few studies have shown that RDW can be used as a predictor of mortality in CAD patients after PCI [38,39,40,41,42,43]. However, previous studies only dichotomize RDW groups. The reported cut-off values ranged from 13.1% to 14.8%, and the studies showed a higher risk of all-cause mortality and CV mortality in high RDW group, in comparison with low RDW groups [38,39,44]. Bao et al. [16] reported a meta-analysis of 12 studies with a total of 17,113 CAD patients undergoing PCI. The study demonstrated the risk ratio was 1.77 for all-cause mortality and 1.70 for CV mortality, comparing the higher RDW group with the lower RDW group [16]. Latif et al. [15] reported another meta-analysis including 21 studies and 56,425 CAD patients undergoing PCI. The results showed that higher RDW had higher in-hospital all-cause mortality (OR 2.41), long-term all-cause mortality (OR 2.44) and cardiac mortality (OR 2.65) [15]. The aforementioned two meta-analyses conducted a quantitative synthesis of smaller studies with high heterogeneity. Different cut-off values of RDW may be potential sources of this heterogeneity. Our research included more than 10,000 patients from three hospitals, with divided RDW into five groups; the result showed a linear and dose–response relationship between RDW and mortality risks. Thus, our study demonstrated clearly that RDW was a robust dose–response predictor of all-cause mortality and CV mortality in CAD patients undergoing PCI.

Our study had some limitations. Firstly, this study consisted of hospital-based research, including one medical center and two regional hospitals in Taiwan. Whether the findings of this study could be extrapolated to other hospital levels, or patients in other countries, still needs to be evaluated. However, NHI and mortality follow-up with approximately 97% [20] participation of the citizens, mitigating the limitations of the hospital-based data source. Secondly, this study did not distinguish between acute and chronic context of coronary artery disease, and could not show whether the two populations had different results. However, this study used multivariable analysis to adjust the diagnosis when admission included acute or chronic coronary artery disease. RDW is still an independent and effective factor related to mortality. Thirdly, this study spanned more than 10 years, and the standard practice of medical care might change overtime. Fourthly, nutritional information in the study population might affect the value of RDW [5]. This study lacked the adjustment of some unmeasured confounding factors, such as iron, folate, vitamin B12 and other nutritional information. Fifthly, this study did not collect information of inflammatory biomarkers such as C-reactive protein [27], procalcitonin [45] or interleukin-6 [28], as these biomarkers were not checked in most of our study subjects. Finally, this study only discussed baseline RDW on admission, and did not include subsequent changes in RDW for analysis.

## 5. Conclusions

We found a linear and dose–response relationship between RDW and all-cause and CV mortalities in patients with CAD undergoing PCI. Thus, RDW may be used as a clinical parameter to predict future mortality and prognosis in patients with CAD undergoing PCI.

## Figures and Tables

**Table 1:** Background Characteristics of Study Participants Stratified by Quintiles of Red Cell Distribution Width.

Groups 1–5 of RDW (%)	All	Group 1 ≤13.3	Group 2 13.4–14.0	Group 3 14.1–14.7	Group 4 14.8–15.8	Group 5 ≥15.9	*p*
Number of patients	10669	5787	2246	1133	779	724	
Age, years	65.4 (12.1)	63.3 (11.6)	67 (12.1)	68.1 (12.3)	69.4 (11.8)	69.1 (12.3)	<0.001
Male	8382 (78.6)	4742 (81.9)	1746 (77.7)	838 (74)	550 (70.6)	506 (69.9)	<0.001
BMI, kg/m^2^	26 (10.6)	26.3 (10.8)	26.2 (11.7)	25.8 (9.4)	25.1 (11.3)	24.4 (4.2)	<0.001
Total number of stents	1.3 (1)	1.3 (1)	1.3 (1)	1.3 (1)	1.3 (1)	1.3 (1.1)	0.057
Hemoglobin, gm/dL	13.5 (2.2)	14.2 (1.8)	13.6 (2.1)	12.7 (2.2)	12 (2.3)	11.1 (2.3)	<0.001
eGFR, mL/min/1.73 m^2^	70.3 (29.9)	77.6 (25.4)	69.2 (28.5)	59.3 (32.8)	56 (34.9)	51.3 (35.1)	<0.001
WBC, 10^3^/mm^3^	6.6 (9.8)	6.3 (9)	6.7 (9.7)	6.6 (9.9)	7.2 (11.1)	8.3 (13.4)	<0.001
Total cholesterol, mg/dL	172.2 (41.9)	174.3 (40.2)	174.6 (40.4)	171.8 (42.1)	167 (50.8)	155.2 (43.1)	<0.001
Triglyceride, mg/dL	147.4 (107.5)	150 (99)	149.5 (124.5)	143 (99.5)	143.1 (136)	132.8 (89.8)	<0.001
Risk factors							
Hypertension	7276 (68.2)	3782 (65.4)	1565 (69.7)	802 (70.8)	589 (75.6)	538 (74.3)	<0.001
Hyperlipidemia	5628 (52.8)	3158 (54.6)	1212 (54)	567 (50)	376 (48.3)	315 (43.5)	<0.001
Diabetes mellitus	4373 (41)	2186 (37.8)	899 (40)	523 (46.2)	377 (48.4)	388 (53.6)	<0.001
Current smoker	721 (6.8)	394 (6.8)	177 (7.9)	71 (6.3)	47 (6)	32 (4.4)	0.0183
Medical history							
Previous MI	1221 (11.4)	608 (10.5)	245 (10.9)	154 (13.6)	99 (12.7)	115 (15.9)	<0.001
ischemic stroke	980 (9.2)	452 (7.8)	229 (10.2)	117 (10.3)	99 (12.7)	83 (11.5)	<0.001
PVD	631 (5.9)	253 (4.4)	118 (5.3)	110 (9.7)	83 (10.7)	67 (9.3)	<0.001
CPD	1342 (12.6)	589 (10.2)	307 (13.7)	179 (15.8)	149 (19.1)	118 (16.3)	<0.001
Previous PCI	2379 (22.3)	1213 (21)	516 (23)	304 (26.8)	179 (23)	167 (23.1)	0.004
Previous CABG	423 (4)	165 (2.9)	83 (3.7)	65 (5.7)	51 (6.5)	59 (8.1)	<0.001
Diagnosis							<0.001
ST-elevation MI	1470 (13.8)	782 (13.5)	318 (14.2)	167 (14.7)	91 (11.7)	112 (15.5)	
Non-ST elevation MI	1369 (12.8)	655 (11.3)	274 (12.2)	163 (14.4)	133 (17.1)	144 (19.9)	
Unstable angina	222 (2.1)	96 (1.7)	51 (2.3)	31 (2.7)	22 (2.8)	22 (3)	
Stable angina	7608 (71.3)	4254 (73.5)	1603 (71.4)	772 (68.1)	533 (68.4)	446 (61.6)	
Type of intervention							<0.001
Angioplasty only	2121 (19.9)	1128 (19.5)	432 (19.2)	242 (21.4)	155 (19.9)	164 (22.7)	
BMS	1878 (17.6)	846 (14.6)	431 (19.2)	243 (21.4)	178 (22.8)	180 (24.9)	
DES	6338 (59.4)	3643 (63)	1313 (58.5)	607 (53.6)	422 (54.2)	353 (48.8)	
Both BMS and DES	332 (3.1)	170 (2.9)	70 (3.1)	41 (3.6)	24 (3.1)	27 (3.7)	
Medications							
Aspirin	5818 (54.5)	3185 (55)	1240 (55.2)	608 (53.7)	416 (53.4)	369 (51)	0.243
Clopidogrel	7583 (71.1)	4116 (71.1)	1589 (70.7)	795 (70.2)	560 (71.9)	523 (72.2)	0.861
Ticagrelor	1162 (10.9)	701 (12.1)	259 (11.5)	93 (8.2)	63 (8.1)	46 (6.4)	<0.001
Nitrates	3397 (31.8)	1846 (31.9)	733 (32.6)	325 (28.7)	259 (33.2)	234 (32.3)	0.156
Amiodarone	435 (4.1)	127 (2.2)	96 (4.3)	71 (6.3)	69 (8.9)	72 (9.9)	<0.001
Beta-blockers	3858 (36.2)	2096 (36.2)	814 (36.2)	399 (35.2)	270 (34.7)	279 (38.5)	0.561
Diltiazem	426 (4)	239 (4.1)	96 (4.3)	52 (4.6)	21 (2.7)	18 (2.5)	0.050
Dihydropyridine CCBs	1564 (14.7)	758 (13.1)	366 (16.3)	186 (16.4)	142 (18.2)	112 (15.5)	<0.001
ACEIs	787 (7.4)	402 (6.9)	165 (7.3)	81 (7.1)	69 (8.9)	70 (9.7)	0.045
ARBs	2722 (25.5)	1476 (25.5)	615 (27.4)	260 (22.9)	195 (25)	176 (24.3)	0.069
Loop diuretics	1128 (10.6)	383 (6.6)	244 (10.9)	188 (16.6)	137 (17.6)	176 (24.3)	<0.001
Spironolactone	513 (4.8)	179 (3.1)	115 (5.1)	76 (6.7)	56 (7.2)	87 (12)	<0.001
Statins	4125 (38.7)	2343 (40.5)	896 (39.9)	385 (34)	279 (35.8)	222 (30.7)	<0.001
OADs	1819 (17)	933 (16.1)	360 (16)	211 (18.6)	158 (20.3)	157 (21.7)	<0.001
Insulin	281 (2.6)	94 (1.6)	56 (2.5)	39 (3.4)	37 (4.7)	55 (7.6)	<0.001
PPIs	1992 (18.7)	852 (14.7)	427 (19)	258 (22.8)	195 (25)	260 (35.9)	<0.001
H2-blockers	407 (3.8)	197 (3.4)	91 (4.1)	57 (5)	28 (3.6)	34 (4.7)	0.056

Abbreviations: ACEI, angiotensin-converting enzyme inhibitor; ARB, angiotensin receptor blocker; BMI, body mass index; BMS, bare-metal stent; CABG, coronary artery bypass graft; CCB, calcium channel blocker; CPD, chronic pulmonary disease; DES, drug-eluting stent; eGFR, estimated glomerular filtration; MI, myocardial infarction; OAD, oral antidiabetic drug; PCI, percutaneous coronary intervention; PPI, proton-pump inhibitor; PVD, peripheral vascular disease; RDW, red cell distribution width; WBC, white blood cell.

**Table 2:** Mortality Outcome of Study Patients stratified by Quintiles of Red Cell Distribution Width.

Groups of RDW (%)	Group 1 ≤13.3	Group 2 13.4–14.0	Group 3 14.1–14.7	Group 4 14.8–15.8	Group 5 ≥15.9	*p*
Number of patients	5787	2246	1133	779	724	
6-month all-cause mortality	71 (1.23)	65 (2.89)	51 (4.50)	60 (7.70)	104 (14.36)	<0.001
6-month CV mortality	35 (0.60)	29 (1.29)	24 (2.12)	19 (2.44)	45 (6.22)	<0.001
1-year all-cause mortality	132 (2.28)	110 (4.90)	93 (8.21)	102 (13.09)	170 (23.48)	<0.001
1-year CV mortality	58 (1.00)	53 (2.36)	40 (3.53)	36 (4.62)	70 (9.67)	<0.001

Abbreviations: CV, cardiovascular; RDW, red cell distribution width.

**Table 3:** Multivariable Logistic Regression Analysis for Mortality Outcome of Study Patients.

	Unadjusted OR	95% CI	*p* for Trend	Adjusted OR	95% CI	*p* for Trend
6-month all-cause mortality						
Group 1 RDW ≤13.3	1.000	-	0.005	1.000	-	0.004
Group 2 RDW 13.4–14.0	2.399	1.707–3.370		1.554	1.090–2.216	
Group 3 RDW 14.1–14.7	3.794	2.632–5.468		1.608	1.089–2.375	
Group 4 RDW 14.8–15.8	6.716	4.722–9.553		2.245	1.523–3.310	
Group 5 RDW ≥15.9	13.501	9.874–18.461		3.241	2.255–4.657	
6-month CV mortality						
Group 1 RDW ≤13.3	1.000	-	0.004	1.000	-	0.022
Group 2 RDW 13.4–14.0	2.150	1.311–3.525		1.423	0.856–2.365	
Group 3 RDW 14.1–14.7	3.556	2.107–6.002		1.605	0.925–2.784	
Group 4 RDW 14.8–15.8	4.108	2.338–7.218		1.441	0.786–2.642	
Group 5 RDW ≥15.9	10.893	6.954–17.062		3.058	1.821–5.137	
1-year all-cause mortality						
Group 1 RDW ≤13.3	1.000	-	0.004	1.000	-	<0.001
Group 2 RDW 13.4–14.0	2.206	1.705–2.855		1.386	1.055–1.822	
Group 3 RDW 14.1–14.7	3.831	2.914–5.036		1.589	1.180–2.138	
Group 4 RDW 14.8–15.8	6.455	4.925–8.459		2.090	1.543–2.830	
Group 5 RDW ≥15.9	13.146	10.305–16.771		3.192	2.398–4.248	
1-year CV mortality						
Group 1 RDW ≤13.3	1.000	-	0.006	1.000	-	0.015
Group 2 RDW 13.4–14.0	2.387	1.640–3.476		1.555	1.053–2.295	
Group 3 RDW 14.1–14.7	3.615	2.404–5.436		1.585	1.027–2.447	
Group 4 RDW 14.8–15.8	4.786	3.136–7.305		1.623	1.023–2.574	
Group 5 RDW ≥15.9	10.572	7.396–15.113		2.850	1.883–4.312	

Abbreviations: CI, confidence interval; CV, cardiovascular; OR, odds ratio; RDW, red cell distribution width.

## Data Availability

The data used in this study were gathered from Integrated Medical Database, National Taiwan University Hospital and health insurance claims of Taiwan National Health Insurance (NHI) Administration and available from the corresponding author upon reasonable request.

## Supplementary Materials

The following are available online at https://www.mdpi.com/article/10.3390/biomedicines10010045/s1, Figure S1: Study flow-chart. Table S1: Multivariable Cox-Proportional Hazards Regression Analysis for Mortality Outcome of Study Patients. Table S2: Multivariable Logistic Regression Analysis for Mortality Outcome according to the optimal cut-point value of RDW.
