# Supplementary material for: Red Cell Distribution Width and Mortality in Patients Undergoing Percutaneous Coronary Intervention
Source: Biomedicines. 2021 Dec 26;10(1):45. doi: 10.3390/biomedicines10010045 (PMC8772904; doi:10.3390/biomedicines10010045)
Supplement: Supplementary file 1 [file biomedicines-10-00045-s001.zip › supplemenatary tables.pdf]

Supplementary Table S1. Multivariable Cox-Proportional Hazards Regression Analysis for Mortality Outcome of Study Patients

	Unadjusted HR	95% CI	*p* for trend	Adjusted HR	95% CI	*p* for trend
1-year all-cause mortality						
Group 1 RDW ≤ 13.3	1.000	-	.019	1.000	-	.006
Group 2 RDW 13.4-14.0	2.166	1.682-2.790		1.394	1.078-1.804	
Group 3 RDW 14.1-14.7	3.681	2.823-4.800		1.592	1.208-2.099	
Group 4 RDW 14.8-15.8	5.973	4.613-7.735		2.003	1.518-2.643	
Group 5 RDW ≥15.9	11.100	8.843-13.934		2.689	2.076-3.485	
1-year CV mortality						
Group 1 RDW ≤ 13.3	1.000	-	.006	1.000	-	.015
Group 2 RDW 13.4-14.0	2.375	1.636-3.446		1.533	1.049-2.240	
Group 3 RDW 14.1-14.7	3.601	2.407-5.387		1.568	1.029-2.387	
Group 4 RDW 14.8-15.8	4.792	3.162-7.264		1.609	1.031-2.509	
Group 5 RDW ≥15.9	10.378	7.328-14.698		2.710	1.825-4.026	

Abbreviations: CI, confidence interval; CV, cardiovascular; HR, hazard ratio; RDW, red cell distribution width.

Supplementary Table S2. Multivariable Logistic Regression Analysis for Mortality Outcome according to the optimal cut-point value of RDW.

	Unadjusted OR	95% CI	Adjusted OR	95% CI
6-month all-cause mortality				
RDW <13.3	1.000	-	1.000	-
RDW ≥13.3	4.931	3.731-6.516	2.021	1.496-2.731
6-month CV mortality				
RDW <13.8	1.000	-	1.000	-
RDW ≥13.8	4.638	3.292-6.533	1.919	1.314-2.802
1-year all-cause mortality				
RDW <13.8	1.000	-	1.000	-
RDW ≥13.8	5.125	4.297-6.112	1.952	1.599-2.384
1-year CV mortality				
RDW <13.8	1.000	-	1.000	-
RDW ≥13.8	4.301	3.313-5.584	1.732	1.293-2.319

Abbreviations: CI, confidence interval; CV, cardiovascular; OR, Odds ratio; RDW, red cell distribution width.
